# Targeting the hallmarks of cancer: the effects of silibinin on proliferation, cell death, angiogenesis, and migration in colorectal cancer

**DOI:** 10.1186/s12906-021-03330-1

**Published:** 2021-05-31

**Authors:** Saba Sameri, Chiman Mohammadi, Mehrnaz Mehrabani, Rezvan Najafi

**Affiliations:** 1 Research Center for Molecular Medicine, Hamadan University of Medical Sciences, Hamadan, Iran; 2 Physiology Research Center, Institute of Neuropharmacology, Kerman University of Medical Sciences, Kerman, Iran

**Keywords:** Colorectal cancer, Silibinin, Angiogenesis, Autophagy, Apoptosis, Migration

## Abstract

**Background:**

Silibinin, as a chemopreventive agent, has shown anti-cancer efficacy against different types of cancers. In the present study, we investigated the anti-cancer activities of silibinin on CT26 mouse colon cell line.

**Methods:**

CT26 cells were treated with different concentrations of silibinin. To examine the cytotoxic effect of silibinin on proliferation, apoptosis, autophagy, angiogenesis, and migration, MTT, colony-forming assay, Annexin V/PI flow cytometry, RT-qPCR, and Scratch assay were used.

**Results:**

Silibinin was found to significantly reduce CT26 cells survival. Furthermore, silibinin strongly induced apoptosis and autophagy by up-regulating the expression of *Bax*, *Caspase-3*, *Atg5*, *Atg7* and *BECN1* and down-regulating *Bcl-2*. Silibinin considerably down-regulated the expression of *COX-2*, *HIF-1α*, *VEGF*, *Ang-2*, and *Ang-4* as well as the expression of *MMP-2*, *MMP-9*, *CCR-2* and *CXCR-4*.

**Conclusions:**

The present study revealed that silibinin shows anticancer activities by targeting proliferation, cell survival, angiogenesis, and migration of CT26 cells.

## Introduction

The transformation of human normal cells into malignant tumors is a multistep process, called carcinogenesis, which requires the accumulation of a variety of genetic and epigenetic aberrations. These hallmarks of cancer include the (i) constant proliferation of tumor cells, (ii) resistance to cell death, (iii) induction of angiogenesisand activation of invasion and (iv) metastasis [1].

One of the most significant hallmarks of cancer is unchecked cell proliferation of cancer cells in the absence of the external stimuli so as to enable them to have unlimited growth, while in normal cells are able to control the number of cell divisions, tissue construction, and function [2]. Furthermore, apoptosis and autophagy, as two main mechanisms of cell death, can be repressed by the cancer cells which in turn leads to uncontrolled proliferation [1].

One more obligatory factor for tumor growth and development is angiogenesis in which the new blood vessels are formed and later lay the ground for metastasis to distant organs [3]. ence, anti-angiogenic drugs have become one of the most promising approaches for cancer treatment. Although many chemotherapeutic agents have been discovered which are effective in blocking the above-mentioned mechanisms, their high toxicities and side effects still remain a critical issue. Thus, exploring other therapeutic approaches with fewer possible side effects is called for. One of such approaches is chemoprevention [4].

Studies have illustrated that many phytochemicals such as silibinin, isothiocyanates, genistein, curcumin and ellagic acid have shown both radiosensitizing and chemosensitizing properties [5, 6]. Silibinin, the major active constituent of Sylimarin, has shown promise against various types of tumors such as prostate, lung, skin, and colon, by targeting the multi-step tumorigenic events such as unrestricted proliferation, loss of cell death, and induction of angiogenesis [7–9]. However, the molecular mechanisms related with the anti-cancer properties of silibinin have not been clearly expounded.

The long period of colorectal cancer (CRC) progression from a precursor lesion, taking about 10 to 20 years, provides a unique opportunity for effective intervention to each step of carcinogenesis [10].

In the present study, we investigated the anti-cancer efficacy of Silibinin on cell proliferation, migration, angiogenesis, apoptosis, and autophagy of CT26 colorectal cancer cell line to lay the foundation for further pre-clinical studies in a mouse model.

## Materials and methods

### Chemicals and cell lines

Silibinin (chemical name: 2,3-Dihydor-3-(4-hydroxy-3-methoxyphenyl)-2-(hydroxymethyl)-6-(3,5,7-trihydroxy-4-oxobenzopyran-2-yl) benzodioxin) was purchased from Sigma-Aldrich. Dimethyl sulfoxide (DMSO), from Sigma, was used to prepare the stock solution of Silibinin. The following working concentration was diluted in the respective mediums and the final concentration of DMSO was < 0.1% (v/v). CT26 Colon Cancer Cell line and VERO cells as normal cell line [11–14] were obtained from National Cell Bank of Iran, Pasteur Institute (Tehran, Iran) and grown in Dulbecco’s modified Eagle’s medium (DMEM) and RPMI-1640 medium respectively, supplemented with 10% fetal bovine serum (FBS; Gibco, Invitrogen) and 100 units/mL streptomycin and penicillin (P/S; Gibco, Invitrogen). According to the standard culture conditions, the air was humified and its temperature was maintained at 37 °C with 5% CO2.

### Cell growth and viability assay

MTT assay was used to determine the growth and viability of cells after silibinin treatments. The CT26 and VERO cells were seeded in 96-plate at the density of 5 × 10^3^ and 6 × 10^3^ cells per well respectively and afterwards incubated overnight, allowing them to attach. Based on the our previous study [15], CT26 cells were treated with different concentrations of silibinin (0–250 μM) and VERO cells with the final concentration of 0–1000 μM of silibinin. Following silibinin treatment for 24, 48 and 72 h, 10 μl MTT was added to each well. After 3 h of incubation at 37 °C, the medium was removed and then 100 μl of DMSO was added to each well to dissolve the resulting formazan product. The absorbance of solubilized formazan was quantified using an automatic microplate reader at 570 nm.

### Colony-forming assay

CT26 cells were treated with the dose of 50 μM of silibinin and after 24 h incubation at 37 °C, they were detached from the plate and reseeded at a density of 400 cells per each well in a new 6-well plate. An additional 7 days of incubation was carried for cells to enable cells to form visualized colonies. The colonies were stained with 0.05% crystal violet solution (dissolved in methanol and water) and subsequently, the number of colonies was counted, using ImageJ software.

### Flow cytometry assay

For investigating the different stages of apoptosis in CT26 cell line, Annexin V-FITC/ propidium iodide (PI) detection kit (MabTag, Germany) was used. After silibinin treatment with a dose of 50 μM, 10^5^ cells were harvested by diluted trypsin and washed twice by PBS. According to the manufacturer’s instructions, the cells were stained and then analyzed, using Attune NxT acoustic focusing cytometer (Life technology, USA) and FlowJo software 10.

### RNA extraction and reverse transcription-polymerase chain reaction (RT-PCR)

Total RNA was isolated from CT26 cells by RNX-Plus kit (Cinnagen, Tehran, Iran) after silibinin treatment with a dose of 50 μM according to the manufacturer’s protocol and the concentration and purity of the resulted RNA was measured by NanoDrop (Thermo Fisher Scientific, Waltham, MA). Afterwards, cDNA was synthesized from the total RNA, using the Takara kit (Kyoto, Japan). The total volume of the reaction mixture for real-time PCR was 20 mL, containing 1 mL cDNA, 7 mL H2O, 10 mL SYBER Blue master mix (Cinnagen, Tehran, Iran), and 1 mL of pmol/mL specific primers. Primer sequences used for real-time quantitative PCR are listed in Table 1. 18S ribosomal RNA (18S rRNA) was applied as an internal control gene for CT26 cells and relative expression was normalized and reported by the 2^-∆∆Ct^ method analysis.

**Table 1:** Primer sequences for qRT-PCR

Gene	Sense strand	Antisense strand
**Bax**	TTTTGCTACAGGGTTTCATCC	TATTGCTGTCCAGTTCATCTC
**Bcl-2**	CTCGTCGCTACCGTCGTGACTTCG	ACCCCATCCCTGAAGAGTTCC
**Caspase-3**	GAATGTCATCTCGCTCTGGTACG	CTGCTCCTTTTGCTATGATCTTCC
**Beclin1**	GGCTGAGAGACTGGATCAGG	GAGAAGCAGCTTCCTGTTCTGG
**ATG5**	TGCATCAAGTTCAGCTCTTCCT	GCAATCCCATCCAGAGTTGC
**ATG7**	TGGCTGCTATTTCTGCAATG	TTCTGGATGCTGCAAAACAG
**Cox-2**	TGCTGTTCCAACCCATGTCA	TCTTGTCAGAAACTCAGGCGT
**HIF-1α**	CCATTCCTCATCCATCAA	CCATCAACTCAGGTAATCCT
**Angiopoeitin-2**	CAGTTCGTTGTTCCGTCTTGTG	CCGTATAGTAATAGTGTCCAGCCATT
**Angiopoeitin-4**	TCCATCCAGTATGAGAAC	GCAGTTATCATTGTCCAT
**CXCR-4**	CATGGAAATATACACTTCGGA	TGCCCACTATGCCAGTCAAG
**CCR-2**	TGTTACCTCAGTTCATCCA	GTTCACCATCATCATAGTCAT
**MMP-2**	CCCACGAAGCCTTGTTTACC	AGCTGTTGTAAGAGGTGCCCTGGAA
**MMP-9**	CCACTAAAGGTCGCTCGGA	GAGTTGCCCCCAGTTACG
**18srRNA**	GTAACCGTTGAACCCCATT	CCATCCAATCGGTAGTAGCG

### Migration assay

For determining the migration ability of CT26 cells, the migration assay was applied. Briefly, in a 24-well plate, 75 × 10^3^ cells were seeded and incubated at 37 °C for 48 h. Next, the culture area was scratched with a crystal pipette tip to make a linear gap in the confluent monolayer and then, to remove the debris, it was washed twice with PBS. Afterwards, each well was treated with different concentrations of Silibinin (0,25,50,75 μM) and then the images of the culture area were taken at 24,48 and 72 h to determine whether cells could fill the generated gap.

### Statistical analysis

SPSS 25.0 software was used for data analysis. One-way variance analysis (ANOVA), followed by Tukey–Kramer pairwise comparison, was performed to determine the significance of the difference between groups. Data were presented as the mean ± standard error (SEM) and the *P*-value of < 0.05 was considered statistically significant.

## Result

### Silibinin inhibits cell growth and proliferation of CT26 cells

**Fig. 1:**
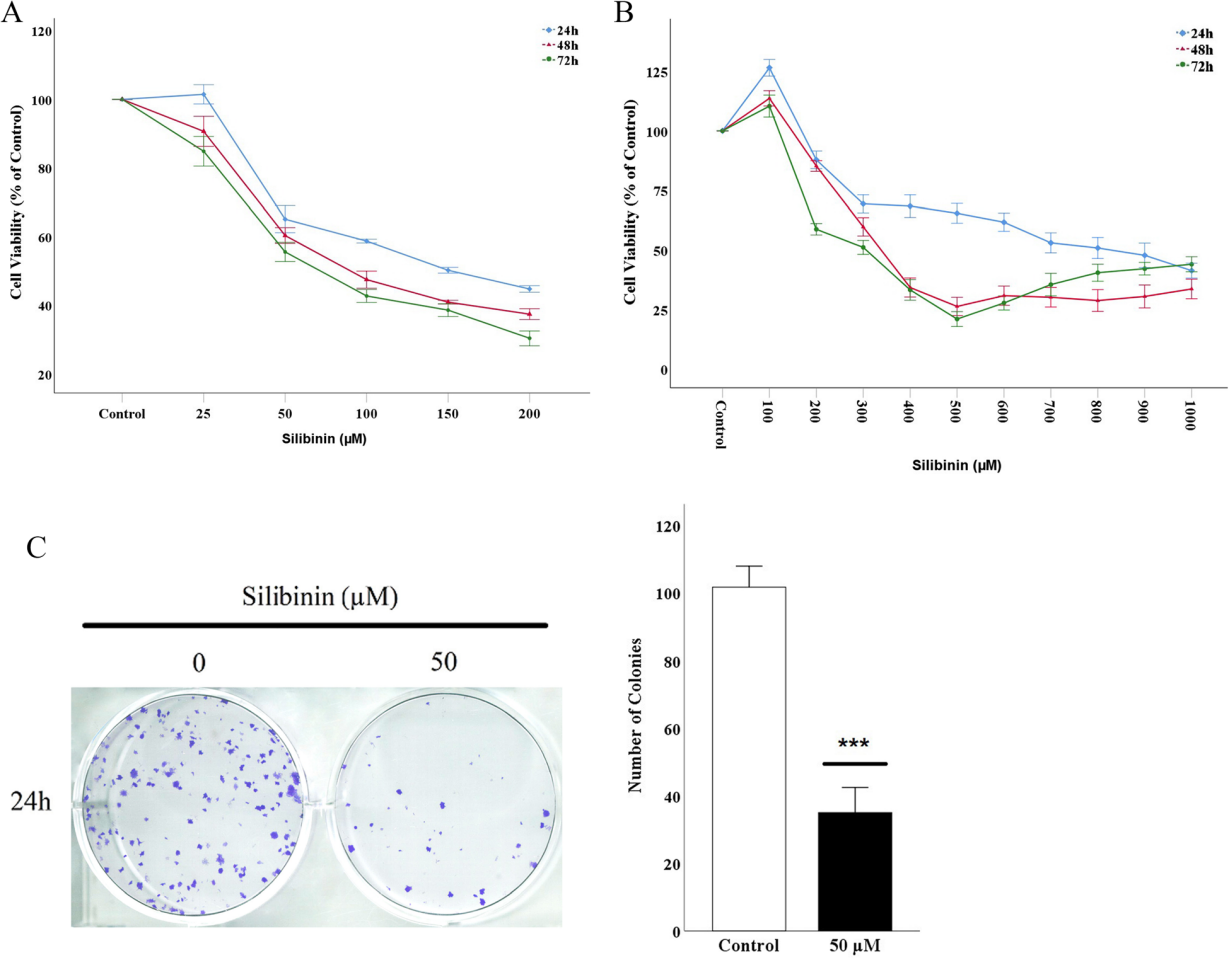
Effect of Silibinin on cell viability. **A**) CT26 and **B**) VERO cells were treated with various concentrations of Silibinin for 24, 48, and 72 h, and their viability was examined by MTT assay. **C**) Representative images and quantitative analysis of colony formation ability of CT26 treated with 50 μM of Silibinin for 24 h. Data are reported as the mean ± standard error of the mean (*n* = 8). *P < 0.05 and ***P < 0.001 compared with control

MTT assay was used to determine the cytotoxic effect of silibinin on CT26 cells treated with different concentration of silibinin for 24, 48 and 72 h. As shown in Fig. 1A, silibinin reduced the cell viability in a time- and dose-dependent manner. As silibinin, in 24 h, reached the IC50 value in approximately 50 μM, this dose was used in further experiments. To determine whether silibinin has toxic effect for normal cell lines, VERO cells were treated with different doses of silibinin (0–1000 μM) (Fig. 1B). The results suggest that silibinin is not toxic for normal cells, showing its specific toxicity against the CRC line. Furthermore, the capacity of cells to proliferate and produce colonies, a key characteristic of cancer cells, was determined by colony-forming assay. The results, which were consistent with the results from the MTT assay, showed a significant reduction in size and number of colonies, treated with 50 μM silibinin, compared to the control group (Fig. 1C). Hence, silibinin suppresses cell growth and proliferation of CT26 cells.

### Silibinin induces apoptosis and autophagic cell death in CT26 cells

**Fig. 2:**
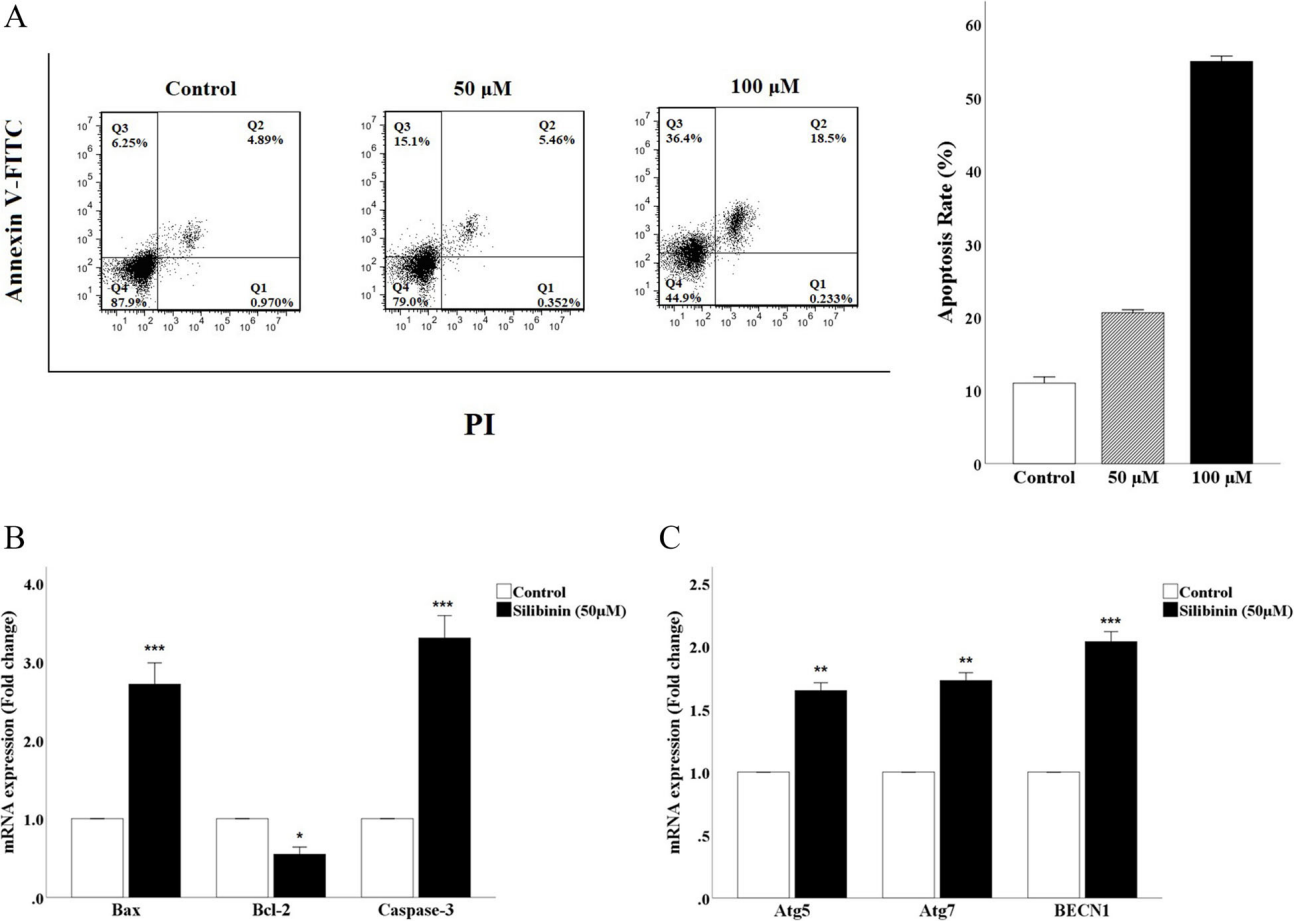
Effect of Silibinin on apoptosis and autophagic cell death. **A**) Representative flow cytometry plots of CT26 cells stained with Propidium iodine/Annexin V-FITC at 24 h after treatment with 50 μM of silibinin. **B**) Effect of silibinin on the expression of Bax, Bcl-2 and Caspase-3 were detected by RT-qPCR. **C**) RT-qPCR analysis of autophagy gene expressions in CT26 cells treated 50 μM of silibinin. Data are represented as mean ± standard error of the mean (*n* = 3). *P < 0.05, **P < 0.01 and ***P < 0.001 versus control group

Flow cytometry was used to detect cell apoptosis in cells treated with silibinin, doses of 0,50 and 100 μM, for 24 h. In the control group, the apoptotic rates in early and late stages of apoptosis were 6.25 and 4.89%, while these rates in the cells treated with a dose of 50 were 15.1 and 5.46% and they were 36.14 and 18.5% for a dose of 100 μM. This clearly indicates that silibinin induced cell apoptosis in a dose-dependent manner (Fig. 2A). Moreover, to find out the mechanisms involved in the apoptosis of CT26 cells, the mRNA expression of *Bax*, *Bcl-2* and *Caspase-3* were investigated in cells treated with 50 μM of silibinin for 24 h, as shown in Fig. 2B. The results showed that silibinin increased the expression of *Bax* and *Caspase-3*, as pro-apoptotic factors, in mRNA levels, while decreased the mRNA expression of *Bcl-2* as an anti-apoptotic factor. These all suggest that cell apoptosis can be accelerated by silibinin through inducing the initiation of mitochondrial apoptosis. As previous studies have demonstrated that silibinin could induce autophagy in several types of tumor cells [16–18], we aimed to investigate the cytotoxic effect of silibinin on autophagy in CT26 cell line. Hence, to confirm the induction of autophagy, a set of related genes including *Atg5*, *Atg7* and *Beclin-1*, which are critical for this process, were studied in this study. We found the mRNA expression of *Atg5*, *Atg7* and *BECN1* in CT26 cells was significantly increased when treated with 50 μM of silibinin for 24 h, compared to the control group (Fig. 2C).

### Silibinin decreases the expression of genes involved in angiogenesis

**Fig. 3:**
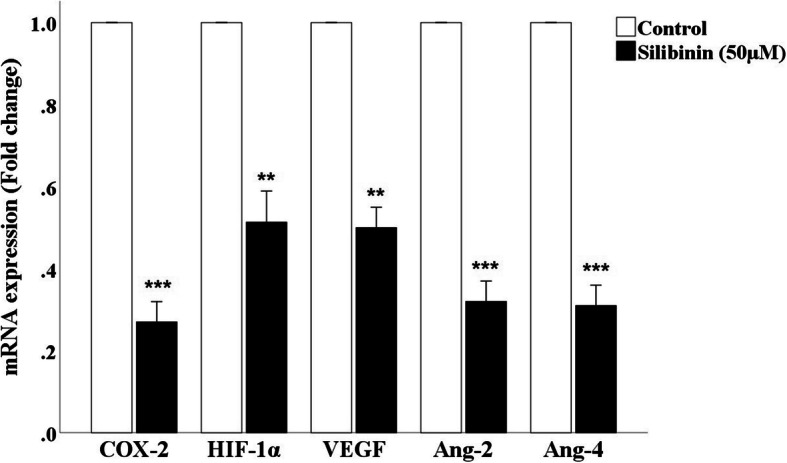
Effect of silibinin on angiogenesis. CT26 cells were treated with 50 μM for 24 h, and the expression of genes was measured by RT-qPCR. Data are represented as mean ± standard error of the mean (n = 3). **P < 0.01 and ***P < 0.001 versus control group

As angiogenesis is one of the main hallmarks of cancer progression, in this study, we aimed to investigate the anti-angiogenic effect silibinin in CRC cells. Thus, quantitative real-time PCR was performed on silibinin-treated (50 μM) and untreated CT26 cells and the results indicated that silibinin considerably declined the mRNA levels of *HIF-1α*, *COX-2*, *VEGF*, *Ang-2*, and *Ang-4*, compared to the untreated group (Fig. 3).

### Silibinin inhibits migration of CT26 cells

**Fig. 4:**
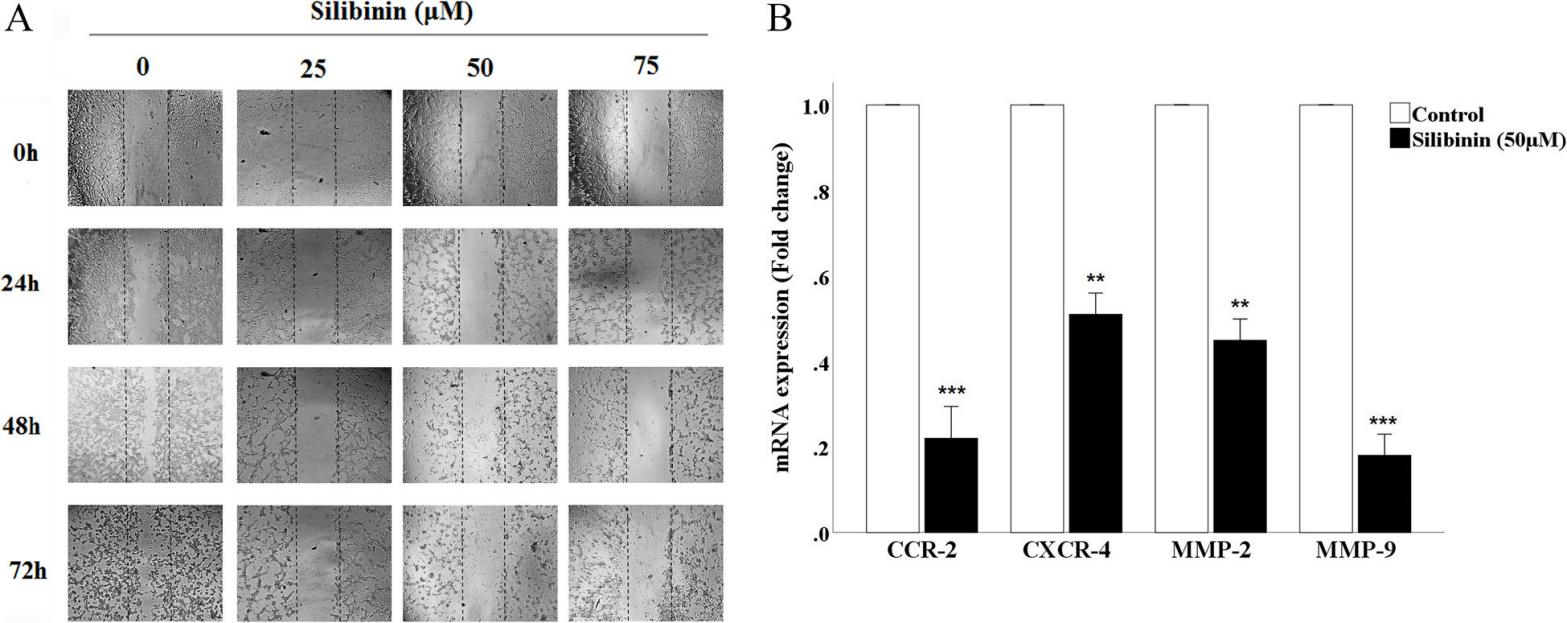
Effect of silibinin on cell migration. **A**) The migration capability of CT26 cells was evaluated by a scratch assay after 0, 24, 48 and 72 h of treatment with different dose of silibinin. **B**) RT-qPCR analysis of MMP-2, MMP-9, CCR-2 and CXCR-4 expression in cells treated with silibinin and untreated cells after 24 h of treatment. Data are represented as mean ± standard error of the mean (n = 3). **P < 0.01 and ***P < 0.001 versus control group

In order to determine the capacity of CT26 cells to migrate, the scratch test was applied. As shown in Fig. 4A, the cells treated with different dose of Silibinin (0, 25, 50 and 75 μM) for 3 consecutive days, showed different levels of migration, suggesting that silibinin could inhibit the migration of the cells in a time- and dose-dependent manner. Furthermore, for detecting the molecular mechanisms involved in the migration, the mRNA expression of *MMP-2* and *MMP-9*, *CXCR-4* and *CCR-2*, were measured in CT26 treated with 50 μM for 24 h. As shown in Fig. 4B, the expression of the above-mentioned genes showed a significant decrease, confirming the results of the scratch test. Thus, it seems that silibinin inhibits the migration of CT26 cells through down-regulation of *MMP-2*, *MMP-9*, *CXCR-4*, and *CCR-2*.

## Discussion

As exhibiting the complete set of hallmarks of cancer is an exclusive characteristic of cancer cells, recent therapeutic approaches have been increasingly aiming to target one or more of these hallmarks. These characteristic have been targeted over the past decade in the development of therapeutic treatments for colorectal cancer as one the most diagnosed and lethal cancers around the world [19]. Silibinin has exhibited anti-cancer properties in different types of tumors [7–9]. In this study we demonstrated the cytotoxic effects of silibinin on four of hallmarks of cancer namely, uncontrolled proliferation, cell survival, induction of angiogenesis and promotion of migration.

Promoting cell death is one effective strategy to overcome unlimited proliferation of cancer cells, as current anti-cancer therapies fail to exert substantial antitumor effects, owing to the aberrant molecular machinery of regulated cell death [20]. Thus, factors capable of inducing both apoptosis and autophagy, as two main pathways of cell death, could be promising candidates for increasing drug sensitivity [21].

Our findings show that silibinin decreased the CT26 cells proliferation in a dose- and time-dependent manner. The antiproliferative and proapoptotic effects of silibinin could be attributed to upregulation of *Bax* as a proapoptotic gene as well as downregulation of anti-apoptotic *Bcl-2* gene. In a study conducted by Yagiet al on the testicular cancer cells a significant increase of *P53* in silibinin-treated cells has been demonstrated [22]. In a similar study, it was found that *ROS*-*JNK*-*P53* cycle was stimulated towards the cell death as well as upregulation of *P53* and *PUMA* expression level and in turn upregulation of *Bcl-2* and *Bax* in cells treated with silibinin [23]. Furthermore, the results of our study show that silibinin considerably increased *Caspase-3* expression, which is consistent with other studies, suggesting that silibinin strongly induced cell cycle arrest and apoptosis through Poly (ADP-ribose) polymerase cleavage, overexpression of *p15Ink4b* and activation of *caspase-3/9* and *JNK* [24–26]. Systemic toxicity, as one of the major concerns in existing cancer treatments, is yet to be resolved. One solution to this issue could be the development of new drugs, having an optimum dose that does not harm normal tissues. Accordingly, the results of this study showed that silibinin appeared to be innocuous to normal cells, as is apparent by its effect on VERO cells [14]. This clearly indicates that silibinin exhibits its cytotoxic property selectively only on cancer cells.

Autophagy, as the second type of programmed cell death (PCD), contributes critically to cellular homeostasis and any disturbances in this process, necessarily, results in interruption of normal cellular functions. The disruption of each stage of autophagy has been seen in many diseases including cancer [27, 28]. It is commonly known that cell death can be a result of cytoplasmic vacuolation caused by excessive levels of autophagy [29]. In the present study, we showed that silibinin induced autophagy by up-regulating mRNA levels of *Atg-5*, *Atg-7*, *Beclin-1*(a necessary factor in early stages of autophagosome formation). Once autophagy is activated, *Atg-7* activates microtubule-associated protein 1-light chain 3-I (*LC3-I*), hence modifying it into its active form (*LC3-II*) [30]. Moreover, *Atg-7* has a crucial role in the formation of *Atg5-Atg12-Atg16L* complex. The exact role of autophagy in cancer progression remains controversial, however most studies suggest that this process acts as tumor suppressor [31]. Similar to our findings, in the study by Jiang et al., the increased levels of *Atg12-Atg5* formation, and *Beclin-1* in MCF-7 cells treated with silibinin, have been observed. In another study on human fibrosarcoma HT1080, it has been shown that silibinin facilitates apoptosis and cell death by inducing autophagy, via Reactive Oxygen Species (ROS) Pathway [32].

Angiogenesis and migration are two interwoven processes in cancer, both of which are highly affected by hypoxia [33]. Cancer cells undergo hypoxia due to unlimited cell proliferation, enhanced metabolism, and abnormal tumor blood vessels. Hypoxia-inducible factor-1 (*HIF-1*), a heterodimeric transcriptional factor, plays a crucial regulatory role by mediating cellular adaptation to hypoxia. This molecule constitutes of two subunits; *HIF-1α* and *HIF-1β*. The overexpression of *HIF-1α* has been observed in many cancers, including CRC [34].

Our findings revealed that silibinin significantly decreased angiogenesis through downregulation of *HIF-1α, COX-2, VEGF, Ang-2* and *Ang-4*. previous studies show that the inhibition of *HIF-1α* and *COX-2* can effectively decrease tumor angiogenesis and growth [35]. Our results is in agreement with the findings of Kim et al., showing that silibinin inhibits generation of *COX-2* through inhibition of the *MAPK (Raf/MEK/ERK*) signal system [36].

*VEGF* has a central role in regulation of angiogenesis, which itself is regulated by *HIF-1α* [34]. Previous studies showed that silibinin caused concurrent decrease of *HIF-1α* and *VEGF* through downregulation of *MAPK (ERK1/2)* and *Akt* signaling [37, 38]. Our findings revealed the decreased level of *HIF-1α* and *VEGF* in CT26 cells treated with silibinin which is likely to have happened through the same pathway.

Moreover, angiopoietins family is among the most important factors in angiogenesis. Angiopoietins are ligands for *Tie2* receptor tyrosine kinases. Of them, *Ang-2* stabilizes the existing vasculature in the presence of *VEGF* and *Ang-4* stimulates the migration of endothelial cell and tube formation [39, 40]. *HIF-1* mediate *Ang-2* expression through having a conserved binding site for *HIF-1*, suggesting that it act as a transcription factor for regulation of *Ang-2* [40]. Furthermore, in 2003, Yamakawa et al., suggested that *Ang-4* can be upregulated by *HIF-1α* under hypoxic condition [41]. Our findings suggest that silibinin probably decreased the *Ang-2/4* mRNA expression through downregulation of *VEGF* and *HIF-1α*.

Regarding the migration, it has been reported that chemokine/cytokine receptors such as *CCR2* and *CXCR-4* play key roles in cell migration and metastatic spread in a variety of cancers, including CRC [42]. Our results showed that silibinin decreased the mRNA expression of *CCR2* which is in agreement with the findings of Forghani et al., on the effects of silibinin on murine breast cancer [43].

Several factors have been identified, having roles in upregulation of *CXCR-4* namely, *HIF-1α* and *VEGF* [44]., A study conducted by Wang et al., on breast cancer cells, introduces silibinin as a novel *CXCR-4* antagonist, inhibiting chemokine ligand 12-induced migration [45], which supports our findings of decreased levels of *CXCR-4* in CT26 cells treated with silibinin.

Furthermore, *MMPs* are among the major factors affecting migration. By possessing the ability to degrade type IV collagen, *MMP2* and *MMP*-*9* facilitate the cell migration and thus metastasis [46]. Interestingly, it has been demonstrated that *MMP-2* and *MMP-9* can be activated by *CXCR4/CXCL12* axis, hence facilitating metastasis [47, 48]. On the other hand, a study carried out by X et al., revealed that the expression of *MMP-2* and *MMP-9* can be induced by *CCL2/CCR2* axis in nasopharyngeal carcinoma metastasis through *ERK1/2* pathways. In line with our findings, it has been reported that silibinin downregulates the expression of both *MMP-2* via *Jak2/STAT3* pathway and *MMP-9* via the *MEK/ERK*-dependent pathway [49, 50].

The protein expression of the genes, investigated in this study were not assessed due to the budget limitation, hence it is recommended that further studies take this under consideration.

**Fig. 5:**
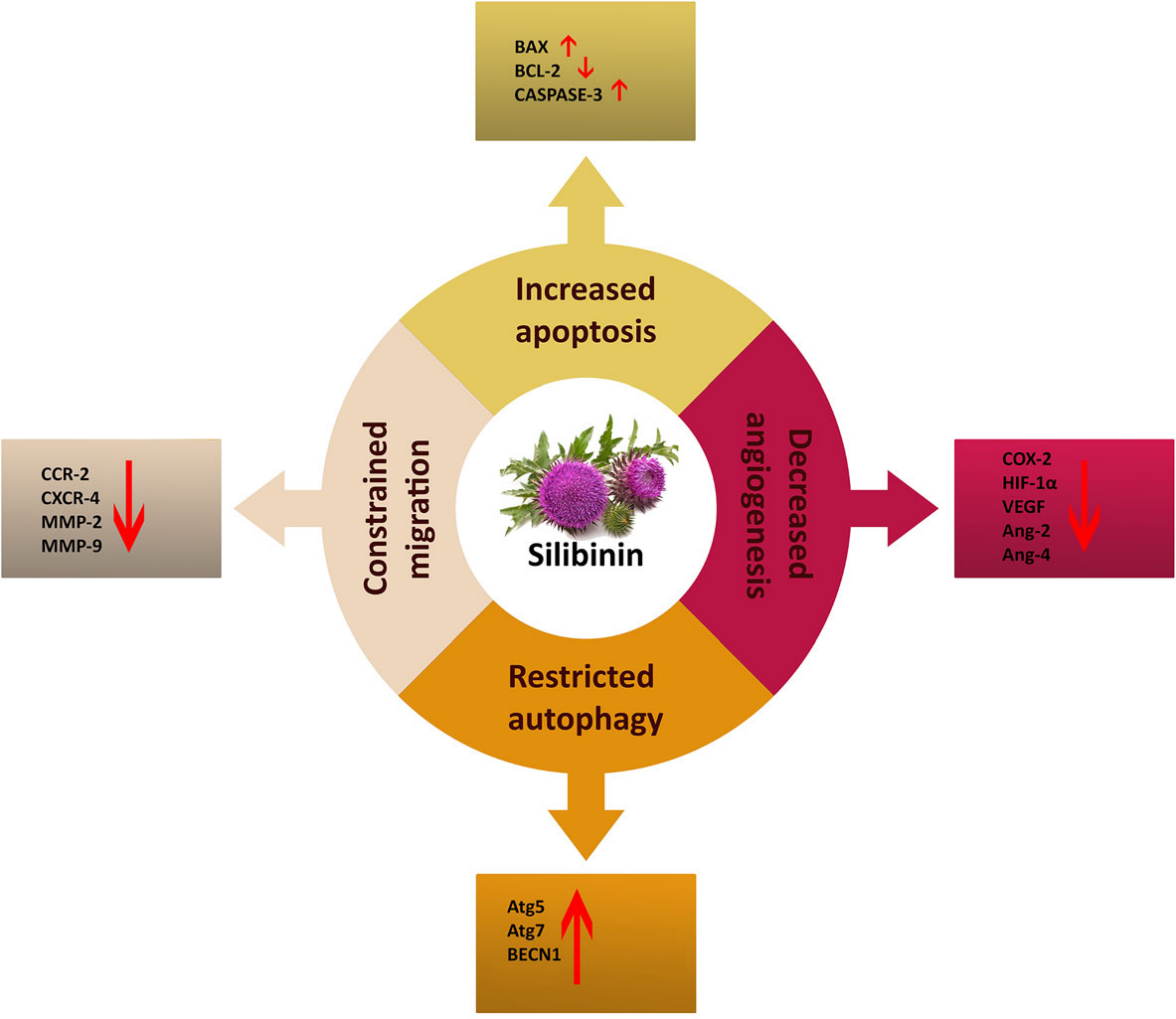
Schematic representation of anti-tumor effects of silibinin on four hallmarks of CRC

In summary, it can be concluded that agents, effecting multi-tumorigenic events exhibit far more promising anti-cancer properties. Silibinin by exerting a wide variety of anti-tumor effects on four of the hallmarks of CRC proves to be a promising potential complementary treatment for existing therapeutic approaches for CRC (Fig. 5).

## Data Availability

The datasets used and/or analysed during the current study are available from the corresponding author on reasonable request.
